# Biological and Psychosocial Factors, Risk Behaviors, and Perinatal Asphyxia in a University Hospital: Matched Case–Control Study, Cali, Colombia (2012–2014)

**DOI:** 10.3389/fpubh.2021.535737

**Published:** 2021-06-21

**Authors:** Javier Torres-Muñoz, Javier Enrique Fonseca-Perez, Katherine Laurent

**Affiliations:** ^1^Neonatal Research Child Health and Development Research Group, Department of Pediatrics, School of Medicine, Faculty of Health, Universidad del Valle, Cali, Colombia; ^2^Department of Gynecology and Obstetrics, School of Medicine, Faculty of Health, Universidad del Valle, Cali, Colombia

**Keywords:** perinatal asphyxia, meconium bronchoaspiration, hypoxic-ischemic encephalopathy, logistic regression model, associated factors, cases and controls matched

## Abstract

**Introduction:** Perinatal asphyxia is one of the main causes of morbidity and mortality in newborns. It generates high costs, both social and economic, and presents modifiable risk factors.

**Objective:** To determine the biological and psychosocial factors and risk behaviors associated with the development of perinatal asphyxia (Sarnat II-III) in newborns from low socioeconomic status in a tier III university hospital in the city of Cali, Colombia.

**Materials and Methods:** With a case and control design, 216 patients were studied (54 cases/162 controls) (1 case/3 matched controls). The cases were defined as newborns with modified or severe perinatal asphyxia (Sarnat II-III) between 2012 and 2014, with gestational age ≥ 36 weeks, with neurological signs not attributable to other causes, multiorgan compromise, advanced reanimation, and presence of a sentinel event. For the analysis, conditional logistic regression models were developed to evaluate association (OR), considering that the cases and controls had been paired by the birth and gestational age variables.

**Results:** The final model showed that, from the group of biological variables, meconium amniotic fluid was identified as a risk factor (OR 15.28, 95%CI 2.78–83.94). Induction of labor lowered the risk of perinatal asphyxia by 97% (OR 0.03, 95%CI 0.01–0.21), and monitoring of fetal heart rate was associated with lower odds by 99% (OR 0.01, 95%CI 0.00–0.31) of developing perinatal asphyxia in the newborn. Regarding social variables, the lack of social support was identified as a risk factor for the development of perinatal asphyxia (OR 6.44, 95%CI 1.16–35.66); in contrast, secondary education lowered the odds of developing perinatal asphyxia by 85% when compared with pregnant women who only had primary school education (OR 0.15, 95%CI 0.03–0.77).

**Conclusion:** Assessment of biological and psychosocial factors and social support is important in pregnant women to determine the risk of developing perinatal asphyxia in a low-income population.

## Introduction

Perinatal asphyxia is one of the main causes of perinatal and neonatal morbidity and mortality, mainly in low-income countries (1, 2). It is estimated globally that in full-term infants, occurrence of perinatal asphyxia is between 2 and 4 per 1,000 newborns; this estimate is higher in pre-mature children (3). Other studies report an incidence of 5–10 per 1,000 live births, with likely underreporting of this entity (4). In Colombia, this incidence is unknown. Perinatal asphyxia can be defined as a syndrome with a wide variety of clinical features in which newborns show specific neurological abnormalities during the first 24 h after birth. This is followed by acute events, characterized by cardiorespiratory depression leading to variable degrees of hypoxemia and hypercapnia and generating metabolic acidosis (5, 6). These events can occur around the time of birth due to fetal lack of oxygen and/or inadequate tissue perfusion (6). Asphyxia and the resulting hypoxic-ischemic encephalopathy are considered frequent causes of chronic disability, such as cerebral palsy, intellectual disability, learning disorders, and epilepsy. It is estimated that ~15–20% of newborns that develop perinatal asphyxia have brain damage and die within the first 28 days of life. Of those who survive, 25% may have permanent neuropsychological sequelae (7, 8). All of this generates deleterious psychosocial effects in society and in the families of affected patients, in addition to high costs for the health system (9, 10).

Perinatal mortality in Latin America varies considerably from one region to another (South America and Mexico with 21.4 per 1,000 live births, Central America with 35.4 per 1,000 live births, and the Caribbean with 52.8 per 1,000 live births). In Colombia, a ratio of 24 deaths per 1,000 live births was identified, and for the department of Valle del Cauca, a ratio of 12.4 per 1,000 live births was identified (11). In the city of Cali, the third most populous city in Colombia, 57% of the total perinatal mortality rate corresponds to the neonatal component, the infant mortality rate (IMR) in 2008 was 13.4 deaths per 1,000 live births, and the neonatal component was of 64% (12). The aforementioned data reflect the magnitude of the problem in the studied population.

## Materials and Methods

### Ethical Considerations

This study was undertaken with approval by the Ethics Committee at the University Hospital del Valle and Universidad del Valle.

Pregnant women who agreed to participate in the study granted authorization through an informed consent, according to Helsinki international ethical regulations.

### Design

This was a retrospective analytical observational study, where cases and controls were matched by the gestational age and the type of delivery (vaginal delivery or cesarean section).

The main outcome was perinatal asphyxia, and independent variables were the maternal factors recorded from the clinical files of the infants and through interviews with the mothers of the patients.

### Study Population

The study was conducted in a tier III hospital in the city of Cali, Colombia, a public institution with facilities for newborn care. In 2012, 5,300 births were attended, with 90% of these subsidized by the healthcare system. The newborns diagnosed with moderate (Sarnat II) and severe (Sarnat III) (13) perinatal asphyxia admitted to the institution, along with the newborns who according to the selection criteria were defined as controls, were considered as the study population. There were three controls for each case.

Definition of cases and controls: For participants, selection criteria and restrictions were applied in a matched case–control design. The criteria were defined in accordance with that established by the American College of Gynecology and Obstetrics and Its Pediatricians (6). The study population consisted of all newborns with gestational age ≥ 36 weeks, diagnosed with moderate (Sarnat II) and severe (Sarnat III) perinatal asphyxia, admitted to the hospital, or all neonates diagnosed with perinatal asphyxia during their stay in the unit, recognized as postnatal asphyxia (Sarnat II or III). Inclusion criteria considered patients with

1. Gestational age ≥ 36 weeks determined by amenorrhea, by early ultrasound, or by Ballard et al. (14)
2. Neonatal neurological signs (convulsion, coma, hypotonic) not attributable to another cause, confirmed by pediatrician evaluation
3. Arterial pH ≤ 7.0 or a base deficit of at least 12 mmol/L taken in the first hour of life
4. An Apgar score of 0–3 after 5 min
5. Multiorgan compromise (two or more organs) not attributable to another cause (central nervous system, renal, pulmonary, cardiovascular, gastrointestinal, metabolic, and hematological)
6. Need for advanced neonatal resuscitation
7. Presence of a sentinel event [uterine rupture, placental abruption, thromboembolism of amniotic fluid, and prolapse of the umbilical cord in a patient who also meets the essential criteria of metabolic acidosis (pH of cord <7 base deficit < -12 meq) and early neurological compromise]

Inclusion criteria for controls are as follows:

1. Not having developed perinatal asphyxia
2. A difference in age from the case of <1 week
3. Being born by the same means of delivery as the cases having gestational age comparable to cases of up to 1 week

The exclusion criteria were defined as follows:

1. Mild perinatal asphyxia in infants (Sarnat I), who developed a clinical spectrum at birth remarkably similar to normal children with sequelae <1%, according to that reported in the literature (15).
2. Patients with major congenital malformations or with congenital or explicable neurological alterations due to a condition other than perinatal asphyxia (electrolyte disturbance, inborn error of metabolism). Such children were not included in the analysis because they were considered possible generators of confusion or information bias. To improve the efficiency of the study, it was necessary to increase the number of controls to three for each case. With the aforementioned and based on the study by Torres-Muñoz et al. (16) from 2010 to 2011, a relationship of three controls was designated for each case.

The sample size was determined considering the prevalence of perinatal asphyxia reported in previous studies (16) with an alpha error of 0.1 and a beta error of 0.20, exposure in the controls of 0.6%, and exposure of the cases of 12.7%. Considering that the cases were paired with controls for two variables (the type of delivery and the gestational age), it was decided to use the comparison formula of proportions for paired groups proposed by Connor in 1987 (17). Pregnant women who agreed to participate in the study signed an informed consent, designed according to required ethical standards. The cases were selected by intentional sampling, when meeting the established selection criteria, during the 2 years (2012–2014) of data collection.

The Medical Outcomes Study (MOS) Social Support Survey evaluated different measurements previously validated in Colombia (18). Questionnaires were administered during follow-up consultations of newborns in the participating institution, guaranteeing the privacy and tranquility of the mothers. Mother self-completed the questionnaires, with support from the care pediatrician prior to delivery who verified that all questions were answered. When a mother was illiterate or could not fill out the questionnaires on her own, she was helped by a pediatrician from the consultation or by the researchers.

The MOS-23 questionnaire is a self-administered form developed by Sherborne et al. (19) in 1991; each item is designed to evaluate

- Structural or quantitative support: 1 item
- Emotional/informational support, as an expression of affection and understanding: 8 items
- Social interaction, such as the availability of other people to meet: 4 items
- Affective support, with real love demonstrations: 3 items
- Instrumental support, material or tangible help received: 4 items

The items are answered in two different ways: four of them are of dichotomous response (yes or no), and the other two are answered according to a four-point Likert scale (0 to 3 points). The total score is obtained by adding the scores obtained in each item, and ranges from 0 to 10 points were considered. The score of this scale was adopted in this research.

### Analysis Plan

To consolidate the information obtained, a form was designed in the Epi Info software version 3, where the data of biological and social variables, obtained through the clinical history of each patient, were entered. In this same form, the MOS questionnaire was also included to facilitate the collection of information and verification of the clinical history, taking advantage of the fact that the mother was present to complete the questionnaire. The data were recorded in an.xls file to be worked in Excel® and then exported to the STATA 13 software (Statacorp Inc. Texas) for statistical analyses.

Biological and social risk factors for the initial evaluation of the quantitative variables were carried out through univariate analysis in which the statistics of central tendency and dispersion were reported, according to the distribution of each variable. For the variables of maternal age, maternal weight, body mass index, and newborn weight, the Shapiro–Wilk-test was used to evaluate normality in the data distribution. Subsequently, a bivariate analysis was performed in which the strength of the association (exposure OR) was determined with respective 95% confidence intervals (95% CIs) between the dependent and independent variables (bivariate analysis), and contingency tables were generated. Regarding the categorical variables, an exploratory univariate analysis was conducted in which absolute frequencies were expressed, followed by a bivariate analysis using the chi-square test or the Fisher exact-test, as appropriate. The strength of the association (OR) and its 95% CI, between the dependent and independent variables, were determined. We determined the respective adjusted OR of each group of variables once the possible effects of interaction and confusion between the different factors and covariables considered were analyzed and discarded. For this purpose, a multiple conditioned logistic regression model was used bearing in mind that it was paired by birth and gestational age. To select the variables included in each of the models, a forward stepwise procedure was used. The conditioned logistic regression was chosen considering that, in this study, pairing was used to control for the type of the delivery and for the gestational age. The conditional logistic regression (CLR) is a specialized logistic regression, so coefficients, odds ratios, and adjustment statistics can be interpreted the same way as for ordinary logistic regression.

## Results

**Figure 1:**
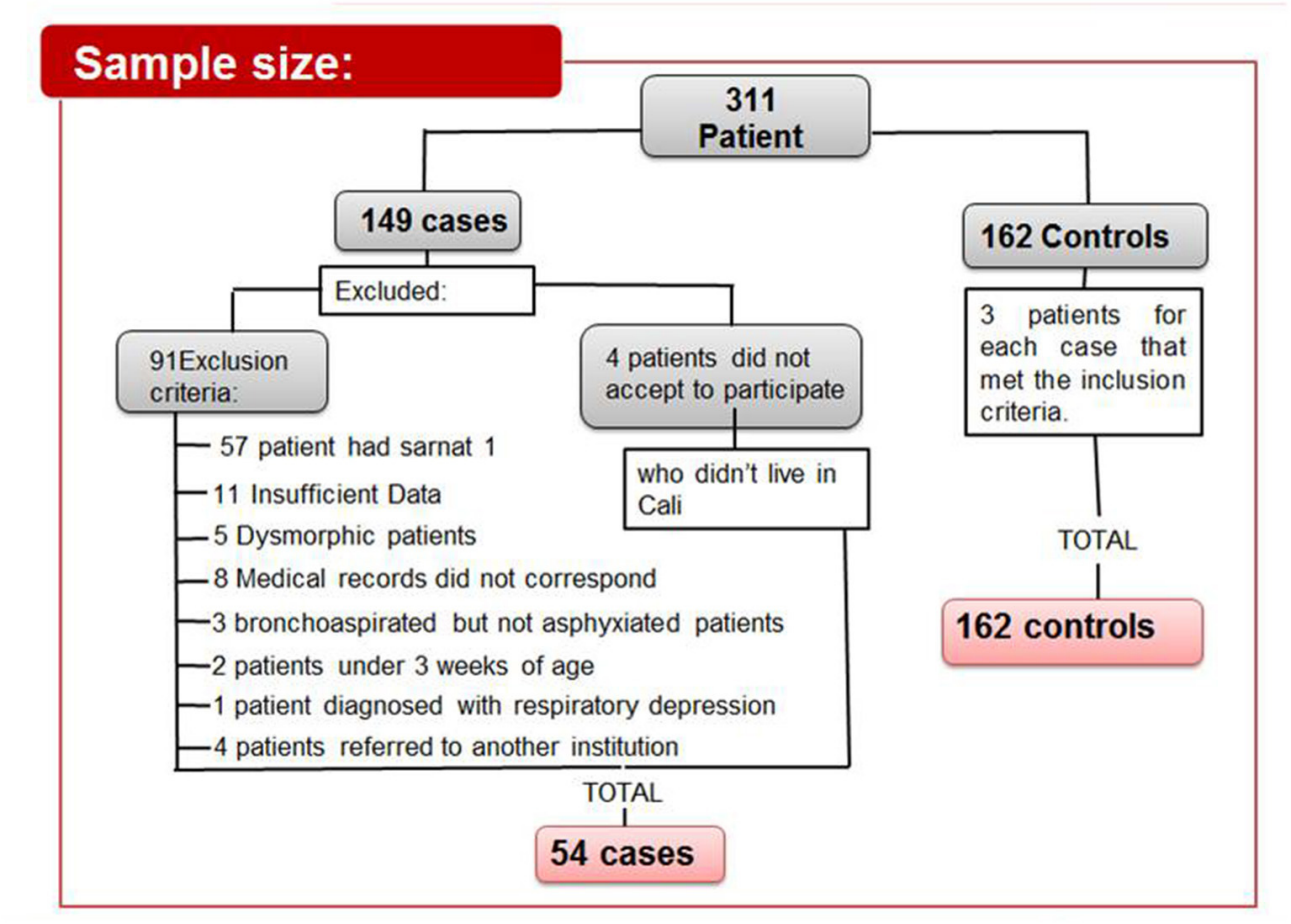
Case selection scheme. Selection process of newborns from cases and controls.

Initially, 311 newborns were admitted, of which 54 cases and 162 controls were selected (Figure 1). The general characteristics of the population in relation to the biological variables are described according to case or control (Table 1). Bivariate comparisons between the cases and the controls showed that not monitoring the fetal heart rate was found more often in the cases than in the controls (*p* < 0.001). Similar results were observed for not monitoring of labor, which was more frequent in the cases than in the controls (*p* < 0.001), and chorioamnionitis (*p* < 0.01). In contrast, oxytocin use was used more often in the controls than in the cases (*p* < 0.001). We observed that newborns considered as cases presented meconium amniotic fluid in 62.30 vs. 13.50% of the controls (*p* < 0.001). Of the babies diagnosed with perinatal asphyxia, 94.40% required resuscitation maneuvers, 90.70% required mechanical ventilation, 96.30% were diagnosed with multiorgan dysfunction, and 5.6% died.

**Table 1:** Description of biological variables.

**Characteristics**	**Cases (54) (%)**	**Controls (162) (%)**	*P*-value^**^
Age of the pregnant patients	24.03	24.7	0.60
Primigravidity	37 (68.50)	73 (45.10)	<0.001
Chorioamnionitis^*^	4 (7.40)	2 (1.20)	0.01
Maternal morbidity	20 (37.70)	50 (32.60)	0.73
Use of oxytocin	17 (32.70)	130 (80.20)	<0.001
Non-monitoring fetal heart rate	13 (24.10)	3 (1.90)	<0.001
Non-monitoring of labor	16 (29.60)	3 (1.90)	<0.001
Meconium amniotic fluid	33 (62.30)	21 (13.50)	<0.001
Gestational age: <38 weeks	17 (31.50)	48 (29.60)	0.80
**Sex:**			
Female	22 (40.70)	73 (45.10)	0.57
Male	32 (59.30)	89 (54.90)	
Cesarean section	20 (37.70)	67 (41.60)	0.61
Neonatal mortality^*^	3 (5.60)	0 (0.00)	<0.001
Alterations of the fetal heart rate: yes^*^	10 (24.40)	1 (0.60)	<0.001
Alterations of labor monitoring: yes	20 (51.28)	14 (8.80)	<0.001

*Cali, Colombia 2012–2014*.

*The general characteristics of the pregnancy and newborn in relation to the biological variables are described, according to case or control*.

* *Chi-square test was performed for variables with frequencies >5, and Fisher's exact-test was used in the opposite case*.

** *Significance level of 0.05*.

Social variables are described in Table 2. Regarding educational attainment, only 48.20% of the cases but 76.5% of the controls had a high school education (*p* < 0.001). In the analysis of subscales with the MOS questionnaires, low support was noted in four categories: emotional subscale (33.30% cases vs. 16.70% controls) (*p* < 0.001); social subscale (22.60% cases vs. 0.60% controls) (*p* < 0.001); affective subscale (37.70% cases vs. 11.70% controls) (*p* < 0.001); and instrumental subscale (42.60% cases vs. 14.20% controls) (*p* < 0.001). In behavioral variables, only two of the cases and one of the controls smoked. Passive exposure to cigarette smoke was present in five cases, and consumption of psychoactive substances was present in three cases and one control. Models of conditioned logistic regression, including the type of delivery and the gestational age, allowed quantifying associations for each group of variables.

**Table 2:** Description of social variables.

**Variable**	**Cases (54) (%)**	**Control (162) (%)**	***P-*value**
Maternal schooling: high school	26 (48.2.0)	124 (76.50)	<0.001
Without a partner	17 (32.70)	42 (25.90)	0.34
Household income: less than the minimum wage	35 (79.50)	84 (54.50)	0.09
Prenatal control: ≤ 3	22 (41.50)	26 (16.10)	<0.001
Unplanned pregnancy	30 (68.20)	59 (38.60)	<0.001
**MOS survey**			
Emotional subscale: poor support^*^	18 (33.30)	27 (16.70)	<0.001
Social subscale: poor support^**^	12 (22.60)	1 (0.60)	<0.001
Affective subscale: poor support^***^	20 (37.70)	19 (11.70)	<0.001
Instrumental subscale: poor support^****^	23 (42.60)	23 (14.20)	<0.001

*Cali, Colombia 2012–2014*.

*Significance level of 0.05*.

* *Emotional subscale: low support <24 points*.

** *Social subscale: low support <9 points*.

*** *Affective subscale: low support <9 points*.

**** *Instrumental subscale: low support <12 points*.

Regarding the odds of developing asphyxia (Table 3), a significant OR was found for several variables. Multiparity (defined as having more than one birth) was associated with the newborn not being suffocated with an odds of 63% (OR 0.37, 95%CI 0.19–0.72). Induction of labor was associated with lower odds of suffocation in 93% (OR 0.07, 95%CI 0.03–0.15). The odds of asphyxia in newborns from mothers with chorioamnionitis was 5.9 times greater (OR 5.9, 95%CI 1.05–33.44); when amniotic fluid had meconium, infants had an 11.7-fold higher risk of developing asphyxia (OR 11.71, 95%CI 5.46–25.14). Cases born from mothers with preeclampsia had a higher odds and had three times greater of developing perinatal asphyxia (OR 3.04, 95%CI 1.51–6.12); in this variable, confounder factors (the type of delivery and the gestational age) were identified, given that women with preeclampsia are more likely to deliver early *via* cesarean section.

**Table 3:** Bivariate analysis between perinatal asphyxia and biological variables.

**Variable**	**Cases (54) %**	**Controls (162) %**	*P-*value^***^	**OR 95% CI**
Multiparity^*^	17 (31.48)	89 (54.94)	<0.001	0.37 (0.19–0.72)
Chorioamnionitis	4 (7.41)	2 (1.23)	0.04	5.95 (1.05–33.44)
Preeclampsia and eclampsia	20 (37.04)	26 (16.25)	<0.001	3.04 (1.51–6.12)
Maternal morbidity^**^	20 (37.74)	50 (32.68)	0.52	1.22 (0.64–2.35)
Use of oxytocin	17 (32.69)	130 (80.25)	<0.001	0.12 (0.06–0.24)
FCF monitoring	41 (75.93)	159 (98.15)	<0.001	0.06 (0.01–0.22)
Monitoring of labor	38 (70.37)	159 (98.15)	<0.001	0.04 (0.01–0.17)
Induction of labor	14 (25.93)	131 (82.39)	<0.001	0.07 (0.03–0.15)
Meconium amniotic fluid	33 (62.26)	21 (13.46)	<0.001	11.71 (5.46–25.14)
Alterations of fetal heart rate	10 (24.39)	1 (0.62)	<0.001	51.93 (6.41–420.4)

*Cali, Colombia 2012–2014*.

* *In this study, multiparity is defined as having more than one delivery*.

** *Maternal morbidity: includes diabetes, hypertension, preeclampsia, and eclampsia*.

*** *Significance level of 0.05*.

Table 4 shows the conditioned OR of the possible explanatory social variables for perinatal asphyxia. Mothers that course secondary education have 68% less chances of having a baby with asfixia (OR 0.32, 95%CI 0.17–0.60), and those with ≥ prenatal visits had 75% lower odds of delivering infants with asphyxia (OR 0.25, 95%CI 0.12–0.51). Regarding the different subscales of the MOS questionnaire, a greater odds of developing asphyxia was observed in the deficient score in the subscales: emotional 2.5 times (OR 2.51, 95%CI 1.25–5.06), social 53.55 times (OR 53.55, 95%CI 6.66–430.05), affective 4.37 times (OR 4.37, 95%CI 2.08–9.20), and instrumental 4.17 times (OR 4.17, 95%CI 2.07–8.38).

**Table 4:** Bivariate analysis between perinatal asphyxia and social variables.

**Variable**	**Cases (54) %**	**Controls (162) %**	*P-*Value^*^	**OR 95% CI**
Schooling: high school	26 (48.15)	124 (76.54)	<0.001	0.32 (0.17–0.60)
With partner	35 (67.31)	120 (74.07)	0.36	0.73 (0.37–1.43)
1–2 legal minimum wage	9 (20.45)	70 (45.45)	<0.001	0.30 (0.13–0.68)
Antenatal visits ≥4	30 (58.82)	136 (85.00)	<0.001	0.25 (0.12–0.51)
**MOS survey**				
Emotional subscale poor support^a^	18 (33.33)	27 (16.70)	0.01	2.51 (1.25–5.06)
Social subscale poor support^b^	12 (22.20)	1 (0.62)	<0.001	53.55 (6.66–430.05)
Affective subscale poor support^c^	20 (37.0)	19 (11.70)	<0.001	4.37 (2.08–9.20)
Instrumental subscale poor support^d^	23 (42.59)	23 (14.20)	<0.001	4.17 (2.07–8.38)
Total classification poor support	22 (40.70)	26 (16.10)	<0.001	3.34 (1.67–6.65)

*Cali, Colombia 2012–2014*.

* *Significance level of 0.05*.

a *Emotional subscale: low support <24 points*.

b *Social subscale: low support <9 points*.

c *Affective subscale: low support <9 points*.

d *Instrumental subscale: low support <12 points*.

In Table 5, after adjusting the model to explain the relationship between the dependent variable asphyxia and all the others, determining if the relationship is significant, the following results were generated: the odds of asphyxia in cases that presented with meconium amniotic fluid was 15.28 times higher compared with that of the controls (OR 15.28, 95%CI 2.78–83.94). Variables related to labor monitoring were also found to be significant in their association with perinatal asphyxia; the introduction of labor decreased the odds by 97% (OR 0.03, 95%CI 0.01–0.21), and the odds or likelihood of not developing asphyxia was lowered by monitoring the fetal heart rate by 99% (OR 0.01, 95%CI 0.00–0.31). Having schooling up to the secondary level decreased the odds of developing perinatal asphyxia by 85% compared to those who only studied until primary school (OR 0.15, 95%CI 0.03–0.77). When applying the MOS questionnaire, the instrumental subscale showed that without adequate support in the cases, the likelihood of developing asphyxia was 6.44 times higher in cases than in those with adequate support (OR 6.44, 95%CI 1.16–35.66). The variables chorioamnionitis, preeclampsia, and <4 prenatal visits were significant in the bivariate analysis but were not significant when entering the final model.

**Table 5:** Multivariate analysis.

**Variable**	**OR**	**Adjusted OR**	*P-*value^**^	**95% CI**
Meconium amniotic fluid	11.71	15.28	0.02	2.78–83.94
Induction of labor	0.07	0.03	<0.001	0.01–0.21
Monitoring of fetal heart rate	0.06	0.01	0.01	0.00–0.31
Secondary education	0.32	0.15	0.02	0.03–0.77
Instrumental subscale^*^	4.17	6.44	0.03	1.16–35.66
Prenatal visits	0.25	0.25	0.13	0.04–1.50

*Perinatal asphyxia with relation to biological and social factors. Cali, Colombia 2012–2014*.

*Multivariate analysis after adjusting the model*.

* *Instrumental subscale: low support <12 points*.

** *Significance level of 0.05*.

## Discussion

The objective of this study was to determine if a relationship existed between psychosocial and biological factors with the development of perinatal asphyxia, which has been demonstrated. The psychosocial and biological factors remained significant after adjusting for different variables. Education higher than primary basic school had a protective effect; on the other hand, low antenatal control and schooling were associated with greater odds for perinatal asphyxia. These results show how social disadvantages were associated with greater risks; their assessment and surveillance in disadvantaged populations may eventually help to reduce current disparities and improve pregnancy outcomes across the socioeconomic spectrum. These results support the previous research published by Ellis et al. (20).

The psychosocial factors have an important impact on the fetal outcome, as proposed by Kramer et al. (21) in a study that identifies the poorest areas as those with the greatest difficulty in access to health services, which reflects one of the dimensions of social exclusion. It is proposed, “that expansion of access and quality of care can have a positive impact on reducing early neonatal mortality.” Therefore, social support has been considered “part of social relationships and refers to the help or assistance provided through interpersonal transactions. It is subdivided into instrumental (provision of tangible help and services), informative (provision of information, advice, or useful suggestions to solve problems), evaluative (provision of useful information for self-evaluation), and emotional (expression of empathy, love, trust, and concern). Social support nurtures the individual from its closest nucleus, which is the family, although there are also sources of external support, such as friends, community, and other actors” (18). The present study evaluated the social support in pregnant women for the development of perinatal asphyxia by using the MOS questionnaire. It was identified that poor social support (inadequate instrumental support) in pregnant women led to a six times greater opportunity of developing asphyxia. Different disciplines have hypothesized that psychosocial factors are related to morbidity and mortality, as in cardiovascular diseases, cancer, and pre-mature birth (22–24).

The biological variables in the final model that were found to be significantly associated with the moderate or severe perinatal asphyxia event were the presence of meconium amniotic fluid, which was associated with a higher odds of asphyxia that is 15 times in comparison to those without it, and the induction of labor and monitoring of the fetal heart rate, which lowered the odds. The presence of meconium in amniotic fluid at the time of delivery has been reported in other investigations; a case–control study in Sweden (25) showed that having meconium amniotic fluid increased by four-fold the development of perinatal asphyxia. In a systemic review, McIntyre et al. (26) reported how the presence of meconium-stained fluid and especially meconium aspiration were strong factors associated with neurological compromise at all gestational ages. The presence of meconium in the amniotic fluid can be part of normal processes or followed by abnormal situations, such as compression of the umbilical cord or uteroplacental insufficiency, considered indicators of fetal distress. Meconium is not always associated with intrapartum problems, morbidity, or neonatal mortality (27). Adverse outcomes have been observed when related to other signs of fetal intolerance at delivery, like late decelerations, increased fetal heart rate, and decreased beat-to-beat variability evaluated through continuous electronic cardiac fetal monitoring. The presence of meconium amniotic fluid is considered a controversial factor, and its importance is permanently a subject of analysis. It is discussed if it can behave as a marker of fetal non-well-being or a direct factor of greater damage (28, 29).

Alterations observed during the monitoring of labor have been shown to be a factor significantly associated with the presence of perinatal asphyxia (25). Without adequate control of the fetal heart rate and monitoring of labor, it is not possible to diagnose high-risk events in time, such as pre-mature placental abruption, meconium fluid, and unsatisfactory fetal status (30). The omission of a partograph during labor was also found to be a factor significantly associated with the presence of perinatal asphyxia (OR 2.25, 95%CI 1.14–4.45). As indicated in a previous study in 2011 (16), in this same center, without proper control and monitoring of labor with an appropriate partograph, it is not possible to diagnose in time two main adverse outcomes such as pre-mature placental abruption and labor with a prolonged expulsive phase. Evidence of an altered fetal heart rate during labor, such as severe and sustained bradycardia, absence of variability, and persistent or tardive decelerations, has been shown to increase up to eight times the likelihood of neurological alterations and asphyxia in newborns (22).

The predictive power of electronic fetal monitoring for the development of neonatal encephalopathy and cerebral palsy is low, and, to date, the use of this monitoring has not decreased the incidence of cerebral palsy (30). Despite this, electronic fetal monitoring is the best tool that obstetricians can use in labor to identify fetal metabolic acidosis and reduce the probability of death or neonatal seizures during labor and delivery (31).

The protective effect of inducing labor, at 97% evidenced in this study, is probably related to the fact that women requiring this management are referred from other centers of less complexity, with a diagnosis of fetal non-well-being, and in conditions that require urgent cesarean section. A similar situation occurs for monitoring of the fetal heart rate that showed a protection of 99% when performed. These factors showed the probability of preventing the event if conditions of adequate and timely access to health services are guaranteed. That is why it is considered particularly important to evaluate the quality of health services, emphasizing on how investing in qualified human resources, as well as physical and technological infrastructure conditions, guarantees and provides adequate care to the mother and child in all levels of care. Thus, these events that result in disability and death can be minimized. With these results, it is possible to state that social and biological conditions in vulnerable populations, such as the one cared for in the center where the study was conducted, have a higher odds of developing perinatal asphyxia due to poor support in the instrumental subscale (MOS survey), low schooling, presence of meconium in the amniotic fluid, lack of labor induction, and inadequate fetal heart rate monitoring.

This study shows the importance of studying perinatal asphyxia as a potential public health problem. It also allows understanding that interventions in biopsychosocial factors, associated with the development of perinatal asphyxia, favor its reduction, particularly in middle- and low-income populations. In our study, maternal education level associated with moderate or severe perinatal asphyxia when pregnant women did not have a primary education. This factor is recognized in other publications as a condition associated with maternal and neonatal morbidity and mortality in low-income countries (32, 33).

Findings of the present study support previous research on the association of meconium amniotic fluid with perinatal asphyxia (2, 25, 26, 28), which, together with perinatal acidosis and alterations in the monitoring of labor, may help clinicians to identify women that require early obstetric interventions. Induction of labor and monitoring of the fetal heart rate, in this research, were associated as factors with a protective effect; the lack of monitoring of maternal and fetal conditions at labor was probably related to the lack of appropriate healthcare infrastructure, equipment, and trained clinicians. Therefore, pregnant women are referred to higher complexity-level institutions where they enter in critical conditions with emergency cesarean requirements or in the final phase of labor, which has repercussions on the health of the newborns.

To achieve the required changes in mortality outcomes and sequelae of this disease, participation from different disciplines and sectors committed to its early identification is required; thus, it is necessary to clearly define groups at risk. According to the results of this study, pregnant women with social risk factors, such as low schooling, inadequate prenatal care, and low instrumental social support, should be included as high priority in the guidelines of our health system in order to improve the quality of their prenatal care.

Regarding the limitations of the study, one of them was the fact of it being retrospective, which favors the presentation of bias, such as memory, during the collection of information in the MOS survey. Another possible bias was the selection of participants, given that most of the population was of low income; hence, they were more likely to be exposed to adverse social and biological conditions, although in the end, no significant differences were demonstrated between the cases and the controls according to household income: less than the minimum wage (*p* < 0.09). Incomplete medical records correspond to 7.3%, which did not significantly affect the analysis.

The strengths of this work highlight the contribution of the validated MOS questionnaire in the results of social variables.

In conclusion, these findings confirm the hypothesis that perinatal asphyxia is related to social and biological factors susceptible to intervention through strategies involving adequate prenatal visits and delivery care. Studies should continue to identify risk factors applicable to our population and early markers of asphyxia that may influence its presentation.

## Data Availability Statement

The datasets generated for this study are available on request to the corresponding author.

## Ethics Statement

The studies involving human participants were reviewed and approved by Ethics Committee of the University Hospital del Valle. Written informed consent to participate in this study was provided by the participants' legal guardian/next of kin.

## Author Contributions

JT-M: design, analysis, and writing of the article. JF-P: analysis and writing of the article. KL: collection of information and writing of the article. All authors contributed to the article and approved the submitted version.

## Conflict of Interest

The authors declare that the research was conducted in the absence of any commercial or financial relationships that could be construed as a potential conflict of interest.
